# Using psychological theory and qualitative methods to develop a new evidence-based website about acupuncture for back pain

**DOI:** 10.1016/j.eujim.2016.05.006

**Published:** 2016-08

**Authors:** Felicity L. Bishop, Maddy Greville-Harris, Jennifer Bostock, Amy Din, Cynthia A. Graham, George Lewith, Christina Liossi, Tim O’Riordan, Rachel Ryves, Peter White, Lucy Yardley

**Affiliations:** a Centre for Applications of Health Psychology, Faculty of Social and Human Sciences, Building 44 Highfield Campus, University of Southampton, Southampton SO17 1BJ, United Kingdom; b Institute of Psychiatry, Psychology & Neuroscience (IoPPN), King’s College London, United Kingdom; c Centre for Innovation & Leadership in Health Sciences, Faculty of Health Sciences, University of Southampton, Highfield Campus, SO17 1BJ, United Kingdom; d Primary Care and Population Sciences, Aldermoor Health Centre, University of Southampton, Southampton, SO16 5ST, United Kingdom; e Zemedia, Bitterne Park, Southampton, SO18 1NH, United Kingdom

**Keywords:** Acupuncture, Back pain, Patient education, Digital intervention, Internet, Qualitative research

## Abstract

**Introduction:**

Potential acupuncture patients seek out information about acupuncture from various sources including websites, many of which are unreliable. We aimed to create an informative, scientifically accurate and engaging website to educate patients about acupuncture for back pain and modify their beliefs in a way that might enhance its clinical effects.

**Methods:**

We used psychological theory and techniques to design an evidence-based website, incorporating multimedia elements. We conducted qualitative “think aloud” audio-recorded interviews to elicit user views of the website. A convenience sample of ten participants (4 male; aged 21–64 years from the local community) looked at the website in the presence of a researcher and spoke their thoughts out loud. Comments were categorised by topic.

**Results:**

The website comprises 11 main pages and addresses key topics of interest to potential acupuncture patients, including beneficial and adverse effects, mechanisms of action, safety, practicalities, and patients’ experiences of acupuncture. It provides information through text, evidence summaries and audio-clips of four patients’ stories and two acupuncturists’ descriptions of their practice, and three short films. Evidence from the think aloud study was used to identify opportunities to make the website more informative, engaging, and user-friendly.

**Conclusions:**

Using a combination of psychological theory and qualitative interviews enabled us to produce a user-friendly, evidence-based website that is likely to change patients’ beliefs about acupuncture for back pain. Before using the website in clinical settings it is necessary to test its effects on key outcomes including patients’ beliefs and capacity for making informed choices about acupuncture.

## 1 Introduction

Acupuncture is a popular form of complementary and alternative medicine [1], [2], [3]. In the UK approximately 4 million acupuncture treatments are provided annually and potential acupuncture patients have more than 10,000 registered acupuncturists to choose from, who practice different acupuncture styles in different settings [4]. So much choice can be challenging for consumers [5] who seek out information about acupuncture from various sources, including members of their social networks, the internet, and other media [6], [7]. These information sources help patients to make choices, for example about which specific acupuncturist to consult [5]. On consulting an acupuncturist, patients typically receive leaflets used to convey information necessary for informed consent, standard versions of which have been published [8]. A review of 401 patient information leaflets about acupuncture used in UK clinical settings found that many leaflets successfully provided information that is ethically sound and consistent with the scientific evidence-base [9]. However, written information leaflets can provide limited amounts of information and can only provide text and static images. In comparison, websites can incorporate additional features that can be more engaging than text (e.g. audio, film) and provide more extensive information to those who are interested without overwhelming others. Indeed, online sources of health information are increasingly important to health care consumers [10], [11], [12], [13] but websites about complementary therapies in particular can be unreliable [14], [15], [16]. A new educational website about acupuncture could therefore be useful to help potential patients make informed choices. Importantly, such a website would differ from advertising by presenting evidence-based information to support individuals making an informed choice to use or not to use acupuncture, rather than encouraging everyone to use acupuncture.

In addition to helping patients to make choices, information about acupuncture might also impact clinical outcomes (particularly if it changes patients’ beliefs). For example, according to the Necessity-Concerns Framework, perceiving one’s medicines to be necessary and having few concerns about them leads people to take medicines as prescribed and thus maximise the intended health benefits [17], [18]. According to Response Expectancy Theory, expecting to experience greater pain relief from an intervention directly leads one to experience greater pain relief [19]. Indeed, evidence suggests that patients’ expectations may impact clinical outcomes [20], [21], [22], [23]. For example, having more positive expectations of acupuncture’s effects predicted lower levels of disability in a 6 month longitudinal cohort study of acupuncture for back pain [23]. Encouraging potential patients to have positive expectations of acupuncture’s effectiveness might thus enhance clinical outcomes. However, developing a new approach based on this idea needs to be done with care for patients and attention to the evidence-base: encouraging unrealistically positive outcome expectancies would be ethically questionable and potentially detrimental to health.

This paper describes the development of an educational website about acupuncture. We aimed to create an informative, scientifically accurate and engaging website that could be used to increase understanding of acupuncture and encourage realistically positive outcome expectancies among people who might be considering using it. We operationalised ‘realistically positive outcome expectancies’ as expectations of benefit that are broadly consistent with the scientific evidence base. We focused on acupuncture for back pain because the beneficial effects of acupuncture for pain are particularly well-documented [24], [25], [26] and patients commonly seek acupuncture for musculoskeletal conditions [4], [27].

## 2 Methods

### 2.1 An evidence-, theory-, and person-based approach

We drew on existing evidence and theory and conducted qualitative research to develop and refine our website using an approach derived from evidence-based, theory-based [28], [29] and person-based [30] intervention development. In the context of a website about acupuncture, we felt this combined approach was more valuable than any single approach. Existing evidence and theory about acupuncture were used to ensure the website content was scientifically accurate. It was important to use existing evidence and theory about health beliefs to communicate information persuasively to an audience who might currently be ignorant or misinformed about acupuncture. A small piece of original qualitative research was designed to ensure our website was engaging and convincing for our target audience.

### 2.2 Planning the intervention

To plan the content and structure of the website we considered four key questions:

1. What psychological constructs are relevant to providing information about acupuncture?
2. What do potential patients want and/or need to know about acupuncture?
3. What is the current scientific evidence-base regarding acupuncture’s effects and mechanisms of action?
4. How should information about acupuncture be provided to most effectively improve patients’ knowledge?

### 2.3 Psychological constructs

Table 1 summarises the psychological constructs that we used when planning our website. Given the popularity of digital health information and the prevalence of unreliable websites about complementary therapies, we decided our website should focus on providing information to help potential patients make informed choices to seek (or not seek) acupuncture. Making an informed choice can be understood as choosing to act in a way that is based on one’s knowledge *and* one’s values [31], [32], [33]. According to this definition, it is incorrect to specify that a particular option is the correct choice for everyone: what counts as the informed choice will differ across equally knowledgeable individuals according to their values. To make an informed choice, a person needs to have an accurate understanding of the options available, have formed an opinion about the options based on their values, and make a decision (or otherwise act in a way) that is consistent with their knowledge and values. An informed choice to try acupuncture requires knowledge about the possible beneficial and adverse effects of acupuncture, a positive attitude to acupuncture, and a decision to try acupuncture. An informed choice not to try acupuncture requires knowledge about the possible beneficial and adverse effects of acupuncture, a negative attitude to acupuncture, and a decision not to have acupuncture. To promote informed choice our website thus needed to improve people’s knowledge of acupuncture. Therefore, knowledge about acupuncture (including both beneficial and adverse effects) was our primary target for change.

Given the potential for beliefs about acupuncture to enhance acupuncture’s effectiveness, our website should also encourage patients to have realistically positive beliefs about acupuncture. While acupuncture research has typically focused on patients’ expectations [20], [21], [22], [23], qualitative research suggests that other beliefs are also important when patients are selecting and evaluating therapies for low back pain, namely: perceptions of credibility, including plausible mechanism of action; concerns about treatment processes and possible adverse effects; and beliefs about the personal suitability of a therapy, i.e. individual fit [34]. Therefore, our secondary targets for change were patients’ expectations about acupuncture outcomes, perceptions of acupuncture’s credibility, concerns about acupuncture, and perceptions of individual fit.

Models of illness behaviour suggest a broader psychological context within which people make informed choices about trying acupuncture. According to the Theory of Planned Behaviour [35], patients’ intentions to try acupuncture are driven by attitudes, subjective norms and perceived behavioural control. Patients will be more likely to try acupuncture if they: believe that acupuncture will benefit them (attitudes), believe that others whose opinions they value would approve of acupuncture (subjective norms), and believe that they control whether or not they try acupuncture (perceived behavioural control). While we did not want to encourage people to try acupuncture (except in the context of an informed choice), we did want to provide information that was consistent with people’s existing cognitive structures or ways of thinking about acupuncture. Therefore, we designed the website to address: the likely consequences (beneficial and adverse) of having acupuncture for the individual (attitudes), other people’s views on acupuncture (subjective norms), and practicalities of trying acupuncture such as affordability and finding a qualified acupuncturist (perceived control).

The Common-Sense Model of illness perception suggests that decisions to try a specific treatment (including complementary therapies) are informed by a patient’s beliefs about that treatment and perceptions of their illness [36], [37], [38], [39]. According to this model, people will be more likely to try acupuncture when they believe that acupuncture is likely to be effective (and have minimal/acceptable side-effects) for their specific condition [17], and they think about their condition in terms of specific dimensions including symptoms, duration, and consequences [40]. Therefore, we included content related to these dimensions when developing material about acupuncture’s effects.

### 2.4 Patients’ information needs

To ensure that we provided information relevant to potential patients we reviewed qualitative literature elucidating patients’ perspectives on acupuncture [5], [6], [34], [41], [42], [43], [44], [45], [46], [47], [48], [49], [50], [51], [52], [53], [54], [55], [56], [57], [58], [59], [60]. We created a framework to map key qualitative findings against those cognitive structures theorised to underpin treatment decisions (expectations, credibility, concerns, individual fit, attitudes, subjective norms, perceived control). This exercise highlighted the need for content to address the following questions: What is it? Will it work? Do the needles hurt? How does it work? Is it suitable for me? What different types of acupuncture are there? What is a good acupuncturist and how can I find one? For example, qualitative studies highlighted specific concerns about acupuncture that we needed to address in the website: cost [48], [49], [51] temporary worsening of symptoms [51] and anticipation of pain/anxiety around needling [34], [42], [44], [52], [55].

To ensure we provided information necessary for potential patients to make an informed choice to try acupuncture we reviewed existing information leaflets and modelled some content on the standard leaflet that was developed through consensus for use in the UK [8]. Informed by these materials we wrote content on common (minor) and rare (serious) adverse effects, safety, and contraindications.

Because our website could be considered (but is not intended) to function as advertising, we were mindful that statements about acupuncture’s effects could be challenged through bodies such as trading standards and the Advertising Standards Authority (ASA) [61]. To prevent this we followed guidance from the Committee of Advertising Practice (CAP), who write and maintain UK advertising codes [62].

### 2.5 Acupuncture’s scientific evidence-base

To ensure we conveyed scientifically accurate information about acupuncture’s effects we consulted systematic reviews [26], [63], [64], [65], major clinical trials [24], [66], safety studies [67], [68], [69], and clinical guidelines [70]. We selected key evidence-based facts about acupuncture to convey in the website including: acupuncture can help relieve chronic pain [26]; acupuncture can relieve pain in patients with chronic low back pain [64]; serious side effects are extremely rare (1 in every 10,000 treatments) [71].

To provide an accessible introduction to the multiple theories of acupuncture we decided to present theories from two major approaches, Western acupuncture [72] and traditional Chinese acupuncture. By presenting two theories we were able to reflect two major approaches to the clinical practice of acupuncture in the UK [73] and appeal to different preferences among potential acupuncture patients. To ensure accuracy and authenticity acupuncturists (authors AD, PW, and GL) trained in each tradition developed this content.

We also drew on qualitative literature to highlight patients’ perspectives on acupuncture. This included (a) additional potential benefits to general wellbeing, such as reduced stress [53], [54], better sleep [41], [53], [54], [55], [56], restoring balance [47], increasing energy [52], and encouraging self-care [46], [47], [51], [57]; and (b) the positive nature of acupuncture treatments themselves as enjoyable [42], [43], [58], [59], relaxing [41], [44], [45], [46], [55], [59], holistic [47], [60], and involving a positive therapeutic relationship [41], [42], [43], [44], [45], [46], [47], [48], [49], [50], [51]. Qualitative and questionnaire literature informed the development of content on needling sensation [44], [74].

### 2.6 Effective information provision

We considered a selection of relevant theories to plan how to provide information effectively to educate people about acupuncture.

We drew on Self-Determination Theory [75] to plan how to design our website so that it would be maximally engaging for people. Self-Determination Theory distinguishes between intrinsic motivation (e.g., curiosity) and extrinsic motivation (e.g., payment) as drivers for action; our website relies on intrinsic motivation as we do not anticipate eventual users to receive external rewards for using it. One part of Self-Determination Theory was particularly relevant: Cognitive Evaluation Theory elaborates on how social contexts can impact on intrinsic motivation, and suggests that intrinsic motivation can be enhanced by satisfying basic human needs of competence and autonomy [75]. Thus, if the website supports people’s perceptions of themselves as competent and autonomous it should enhance intrinsic motivation and be more engaging. To promote perceptions of competence among a target user group who might have limited digital literacy we used simple and consistent navigation. To promote perceptions of autonomy we allowed users as much freedom of choice as possible, for example using a flat menu structure that permitted people to view pages in any order.

Educational theory suggests that people have different learning styles [76]; therefore, we decided to use a variety of formats to present information: written text, photographs and images, audio-clips, and film. We also considered who to attribute different sources of information to. According to Social Learning Theory when we identify with another person (a ‘model’) and perceive them to be competent and similar to us we may learn from observing them [77], [78], [79]. Therefore we decided to use actors of various ages, genders, and ethnicities to narrate first-person accounts of patients’ experiences of receiving acupuncture. We drew on qualitative studies of patients’ experiences of acupuncture to develop the first-person accounts [5], [6], [34], [41], [42], [43], [44], [45], [46], [47], [48], [49], [50], [51], [52], [53], [54], [55], [56], [57], [58], [59], [60].

While our focus was on educating people by providing accurate information about acupuncture, we also wanted to change people’s attitudes towards acupuncture. There are two routes, central and peripheral, to attitude formation and change [80]. According to the Elaboration Likelihood Model, when attitude formation and change occurs via central routes highly-motivated individuals engage in an effortful way with substantive messages, assessing new information in relation to previously-held beliefs, and coming to a reasoned conclusion [81]. According to the Heuristic Systematic Model of persuasion, when attitude formation and change occurs via peripheral routes individuals use simple heuristics or ‘rules of thumb’ based on superficial cues such as source credibility and number of arguments presented [82], [83]. To encourage the development of more informed attitudes towards acupuncture we described scientific evidence to support our substantive messages about acupuncture’s beneficial and adverse effects, presented multiple theories about acupuncture’s mechanisms of action, and bolstered the credibility of the message source by describing the website authors’ and quoted acupuncturists’ credentials.

Guidance for developing patient-focused health information highlights five key issues—information needs, accessibility, quality, readability and comprehensibility, and usefulness [84]. To ensure we addressed different needs for information, we allowed readers to access basic or more complex information as click-throughs. We provided basic information about acupuncture’s effects, then offered a click-through to a more detailed evidence summary describing a specific study or review, and then offered another click through to access the actual scientific paper. To provide access to scientific evidence we wrote accurate text, based on peer-reviewed articles and systematic reviews, and provided an evidence summary and access to a few full papers. We considered colour-blindness and dyslexia in our choice of colours and formatting (e.g., using clear plain text and consistent formatting throughout). We also ordered the items on the pages to make them more accessible for people using text-reader software. To ensure we provided high quality information we used peer-reviewed research. To enhance readability we wrote in short sentences with simple sentence construction. To enhance usefulness we provided information on relevant topics (according to the literature on patients’ experiences of acupuncture), included patient representatives and acupuncturists on the research team, and conducted a think aloud study to elicit users’ feedback.

### 2.7 Integrating diverse theories and evidence

**Fig. 1:**
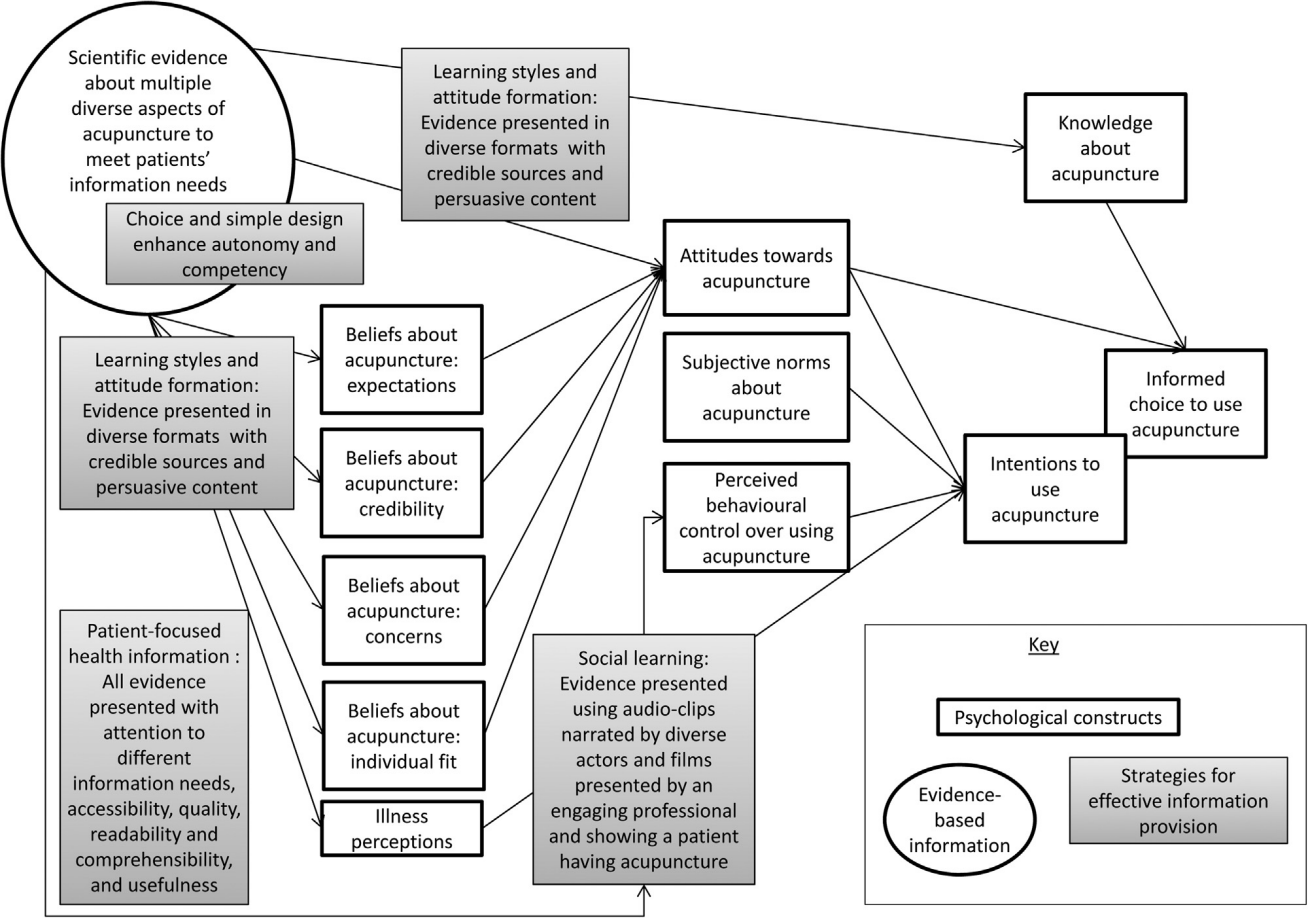
Simplified logic model illustrating how effective evidence-based information provision can impact psychological constructs.

In summary, the process of planning the website was complex and involved drawing on diverse theories and different types of scientific evidence. Fig. 1 provides a simplified illustration of the integration of these theories into a logic model of how our website could influence patients’ informed consent and treatment beliefs. According to this model decisions to use acupuncture occur within a broader psychological context of knowledge, attitudes, and beliefs about acupuncture. The evidence-based information provided on the website was designed to address patients’ information needs given this psychological context. The selected strategies for effective information provision were used to ensure the information provided would influence the intended psychological constructs.

### 2.8 Designing and building the intervention

We used the insights gained during the planning phase to write the content and map out the initial structure of our website using PowerPoint slides. Content was based on published evidence, as described above. To ensure content was relevant to our primary targets for change (knowledge, informed choice) and mapped onto relevant cognitive structures (attitudes, subjective norms, perceived behavioural control, treatment beliefs), we reviewed each draft page for relevance to these targets and structures (see Table 2). We then built the website using LifeGuide (www.lifeguideonline.org), open source software to facilitate the design and scientific testing of web-based behaviour change interventions [85].

Audio-clips were produced for first-person narratives about having acupuncture. Four clips were produced to describe patients’ experiences of the benefits of acupuncture for back pain; the process of acupuncture treatment; needling sensation and minor side-effects (small bruises, bleeding, dizziness); having acupuncture in a clinical trial. Two further clips were produced, each featuring an acupuncturist briefly describing the theory underpinning their approach (Western acupuncture or traditional Chinese acupuncture) and what they would do in a typical consultation with a new patient. The clips were voiced by men (2 patients, 1 practitioner) and women (2 patients, 1 practitioner) including young, middle-aged and older adults.

Three short films were scripted and produced: acupuncture needles; acupuncture treatments; and how acupuncture works. Film is a visual medium that lends itself well to linear narrative and provides an unrivalled, vivid view of the world that can capture the rich detail of events, people and performances. In writing our scripts, we focused on six key considerations for instructional films: the ‘hook’ (an element which captures viewers’ attention), asking questions, synergy between image and narration, clarity of argument, audio/visual cues to denote changes, and argument consolidation [86]. Our three films addressed these considerations by using a professional presenter, interviews, live action, and animated infographics and narration to reinforce key messages. We also considered how to engage viewers on an emotional level, as encouraging viewers to empathise can enhance interest and retention [87]. For example, in productions that include the spoken word viewers may prefer and empathise more readily with conversational language than formal speech [88]. Therefore, we employed an engaging presenter and live action sequences to encourage viewers to imagine themselves in similar situations, and scripted simple, jargon free narration to ease comprehension and reduce cognitive load.

In devising the main messages to be conveyed by the films, we were cognisant that people can store approximately four discrete ‘chunks’ of information in short term memory [89] and that a 30-min video may comfortably elucidate three essential points in some detail. Each of our three films ran for less than 4 min and so together the scripts were written to focus on two key points: acupuncture is a safe and not unpleasant experience, and acupuncture is an established treatment with significant, measurable and positive effects on health.

The acupuncture needles film (duration 2 min 21 s) was designed to alleviate viewers’ concerns about needling and showed a presenter talking to an acupuncturist and being needled (at point Large Intestine (LI) 11). The presenter reacted to needle insertion naturally (with a close-up on her facial expression) and said “Well I can feel something. But it’s not unpleasant—Just a tiny tingling sensation” which was then validated by the acupuncturist who confirmed that “Yes, a lot of people feel it like that. Some people say it’s like a sharp pinch, or a dull ache, or even like a tiny electric shock—there’s quite a variety of opinion.” The presenter thus acted as a credible model for viewers who could then learn vicariously that needling does produce physical sensations but these are typically transient and not painful, thus reducing anticipatory anxiety [90]. The acupuncture treatments film (duration 3 min 23 s) showed a credible model experiencing a full acupuncture treatment and was designed to alleviate viewers’ concerns and render the process less mysterious, thus reducing apprehensiveness or anxieties about seeking an unfamiliar treatment. In this film, elements of a complete acupuncture session were shown in edited form, including the acupuncturist greeting and taking a history from a man with chronic back pain, needle insertion, relaxation, and needle removal; the acupuncturist and patient are then interviewed briefly about the experience. The how acupuncture works film (duration 1 min 59 s) used animations and voice-overs to simplify complex information and educate viewers about traditional Chinese and Western theories about acupuncture’s mechanisms of action.

### 2.9 Think aloud study

A small qualitative study informed the final content and structure of the website. Ethical approval was obtained from the host institution (ergo id: 10933) and all participants gave a priori written informed consent. Posters and online advertisements were used to recruit 10 participants from the host institution and local community (4 male; 4 staff, 4 students; aged 21–64 years; 4 with back pain; 3 had previously received acupuncture). They worked through the website in the presence of the researcher, speaking aloud their thoughts and answering specific probing questions (e.g. “what do you think this page is about?”, “What did you think about navigating through the site?”). Interviews lasted between 18 and 50 min (mean = 35 min) and were audio-recorded. Interviewees’ comments were categorised according to the topic to which they referred and the website was modified accordingly. Interviews proceeded iteratively, with early interviews informing changes to the website which were then presented in later interviews.

## 3 Results

Comments from participants in the think aloud study highlighted aspects of the website that participants found interesting and engaging as well as occasional misunderstandings, confusions and off-putting features. Elements of content, style, and navigation were therefore modified to improve participants’ experiences of using the website. Table 3 presents selected quotes from participants, illustrating how their perspectives were used to suggest changes to the website. Content changes included: adding descriptions of typical consultations; defining some technical terms such as multi-bed clinic and removing others such as meta-analysis; expanding on how to find a qualified acupuncturist (providing more details about acupuncture qualifications and regulation); providing summaries (e.g., a table directly comparing western and traditional Chinese acupuncture styles, a key facts page); providing more details about the professional background of the two acupuncturists to enhance their credibility. Stylistic changes included: ensuring consistent alignment and fonts throughout as participants noticed inconsistencies and saw them as unprofessional, reducing the credibility of the website; using stills from the films as illustrations to make pages more visually engaging; and highlighting team members’ names and qualifications on the ‘meet the team’ page describing study personnel. Navigational changes included: adding a menu bar to all pages to make each page accessible from any other page; page buttons changing colour once viewed. Participants also suggested some desirable changes that we were not able to implement as they could bias the results of the planned evaluation, for example adding links to external websites (e.g. British Acupuncture Council).

**Fig. 2:**
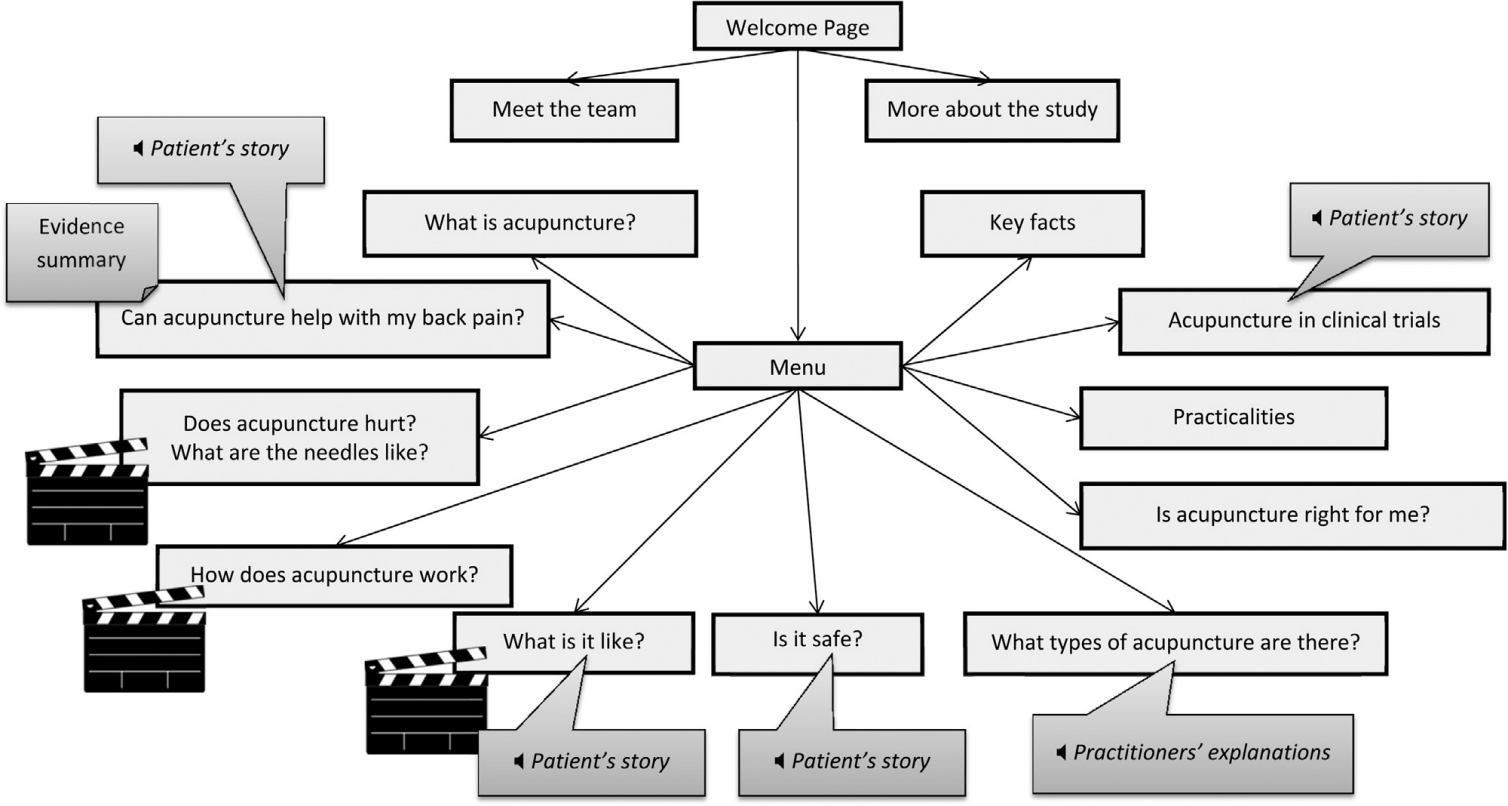
Overview of structure and contents of website.

**Fig. 3:**
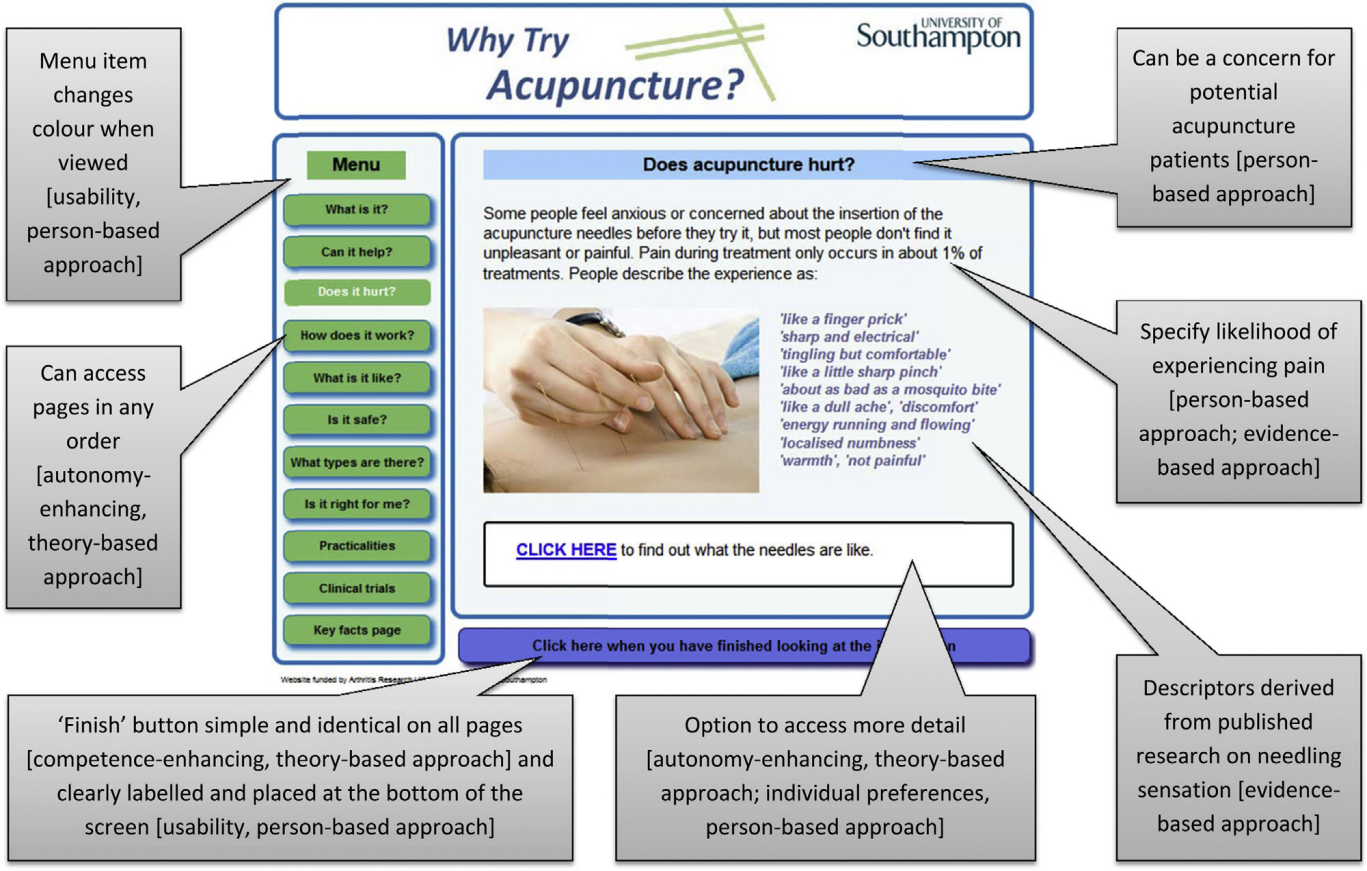
Example page annotated to highlight key features and their development using evidence-, theory-, and person-based approaches.

Fig. 2 illustrates the final structure and main content of the website. Ten topics and key facts are covered across eleven main pages, which can be accessed in any order. The website provides information about: what acupuncture is; benefits and side-effects of acupuncture; acupuncture needles; mechanisms of action; the experience of having acupuncture; safety; Western and Chinese acupuncture styles; contraindications; practicalities (regulation, qualifications, costs, public sector acupuncture); and having acupuncture as part of a clinical trial. Text and images are supplemented by a scientific evidence summary, six audio-clips with photos and transcripts of patients’/practitioners’ experiences, and three films with transcripts. Fig. 3 shows one page (“Does acupuncture hurt?”) and is annotated to illustrate the contribution of evidence-, theory-, and person-based approaches. Additional screenshots from the website are available in supplementary material.

## 4 Discussion

We have developed a new website which conveys scientifically accurate information in a way that should engage members of the public and enable them to make informed choices about acupuncture. The development process drew on the evidence-base for acupuncture’s effects and mechanisms of action, theory about education, treatment-seeking, and motivation, and qualitative research to maximise the website’s likely effects.

Strengths of this project include the multidisciplinary team and the combination of evidence theory and person-based approaches. Team members contributed expertise in acupuncture, digital interventions, film media, and chronic pain, and represented diverse professions including psychology, general practice, physiotherapy, acupuncture. Two team members are patient representatives and their input helped ensure our website addressed important issues and was accessible but not patronising. By drawing flexibly on a combination of approaches we were able to incorporate evidence, theory, and users’ perspectives throughout the intervention development process.

The main limitation is the homogeneous sample of participants in the think aloud study. Involving a more diverse community-based sample would have allowed us to better match the website design to the needs of the general population. A more diverse sample might have uncovered additional issues and/or misunderstandings about acupuncture and suggested further modifications to the website. Future think aloud studies for developing web-based information would benefit from sampling diverse participants from the population of likely end-users. Unfortunately we did not collect data on participants’ ethnicity. In addition, our work may have benefitted from more fully implementing the person-based approach [30]. We drew on existing qualitative research and conducted our own think aloud interviews to elicit users’ perspectives, thus implementing the first major element of the person-based approach. However, we did not implement the second major element, which involves writing ‘guiding principles’ for creating our website [30]. Guiding principles could have helped us to more efficiently prioritise changes from the think aloud study to ensure our content was addressing our aims and fully meeting the needs of our target audience [91].

Eventually our new website could be made available to the general public, used by GPs or other practitioners to help patients make choices about seeking acupuncture, used by acupuncturists as a resource for new patients, and/or adapted to inform clinical trial participants about acupuncture [92]. Ultimately this website has the potential to shape patients’ experiences of acupuncture by modifying their knowledge and beliefs when first initiating treatment. Making an informed choice to try acupuncture and holding realistically positive beliefs about it at the start of treatment could set patients on a positive road to recovery, potentially improving attendance at appointments, engagement with recommended lifestyle changes and self-management, and clinical outcomes. The essential next step is to evaluate the website to determine its effects on patients’ knowledge, informed choice, and beliefs. Such an evaluation is underway and will be reported separately.

## 5 Conclusions

We have developed an evidence-based website that incorporates theory-based techniques to inform members of the public about acupuncture. Before making the website available to the public and/or healthcare professionals, it is necessary to test its effects on patients’ knowledge and beliefs.

## Conflict of interests

The authors declare no competing interests.

## Figures and Tables

**Table 1:** Summary of psychological constructs used to guide website planning.

Theory	Brief definition	Constructs
Informed choice	Making an informed choice to use an intervention is based on one’s knowledge and values.	• Choice to use acupuncture • Knowledge about acupuncture • Attitudes towards acupuncture
Theory of planned behaviour	Patients use acupuncture when they have strong intentions to do so (behavioural intentions) and when they are able to access it (perceived behavioural control). Intentions to use acupuncture are determined by a combination of attitudes towards acupuncture, perceptions of social norms related to acupuncture (subjective norms), and ability to access acupuncture (perceived behavioural control).	• Intention to use acupuncture • Attitudes towards acupuncture • Subjective norms about acupuncture • Perceived behavioural control
Treatment beliefs	Patients will be more likely to use acupuncture if they hold positive beliefs about it. Patients hold beliefs about treatments for low back pain that are structured along four core dimensions: expectations about outcomes, perceptions of credibility, concerns, and perceptions of individual fit.	• Expectations about acupuncture outcomes • Perceptions of acupuncture’s credibility • Concerns about acupuncture’s side-effects • Perception that acupuncture is right for the individual
Common-sense model of illness perception	Patients will be more likely to use acupuncture if they think it is right for them and their particular condition. Patients conceptualise their condition along known dimensions of ‘illness perceptions’, including symptoms, duration, and consequences.	Illness Perceptions: • Perceived symptoms of back pain • Perceived duration of back pain • Perceived consequences of back pain

**Table 2:** Mapping webpages against psychological constructs.

Page	Psychological construct							
	Knowledge	Attitudes	Subjective Norms	Perceived Behavioural Control	Expectations	Credibility	Concerns	Individual fit
What is acupuncture?	✓					✓		
Can acupuncture help with my back pain?	✓	✓			✓			
Does acupuncture hurt?	✓	✓					✓	
How does acupuncture work?	✓					✓		
What is it like?	✓	✓			✓	✓	✓	
Is it safe?	✓				✓		✓	
What types of acupuncture are there?	✓					✓		
Is acupuncture right for me?	✓				✓			✓
Practicalities	✓			✓				✓
Acupuncture in clinical trials	✓					✓		
Key facts	✓	✓		✓	✓	✓	✓	✓

✓ indicates material on this page targets the psychological construct.

**Table 3:** Illustrative quotes from participants used to refine the website.

Topic	Quote	Modification
Content: Acupuncture’s beneficial effects for back pain	So it tells me that acupuncture has been used in the past with back pain, and it gives me the option to read the research to find out and not just believe what's on the page. And then there's a summary saying that […] acupuncture was more effective than placebo or than sham acupuncture which I hadn't even really considered as a thing before so it's quite nice to see that straight away. (Participant 1)	None needed
Content: Patients’ stories	I think it’s good that there are personal stories and personal testimonials because it just makes it a bit more realistic for the user to think well if it helped them then it could help me and he had a nice trusting sounding voice. (Participant 3)	None needed
Content: Practicalities	I think it’s good how you’ve got about how to find a reputable one. Maybe just more about how to find your local acupuncture person? (Participant 10)	Expand on Practicalities − finding a qualified acupuncturist
Content: Jargon	So what exactly is a multibed clinic? ‘Multibed clinics are an affordable option’. So I would want to understand what a multibed clinic was. You know I have a vision of several people laying on one large sofa. Needle for you needle for you. (Participant 5)	Define multibed clinic
Content: Acupuncture styles	You could have a summary, bullet-point of each approach at the end, a comparative table, because it is quite a long thing to read if somebody just wants to see this is two approaches, traditional approach, western approach and then the differences, just like in a brief little table. (Participant 2)	Add table comparing Western and Chinese acupuncture
Style: Alignment	Actually something that I though was maybe the border is not lined up perfectly but then I’m I might be slightly OCD about stuff like that. But then the left one is lined up and if they are meant to be lined up the left one is lined up in a different way to the right one. (Participant 6)	Correct misalignments
Navigation: Menu	What I would say is to continuously have to go back to one main page is a bit of a chore so for the layout to have a menu at the side or a drop-down box at the top would just make it a bit easier for the usability but, as it is, it is very easy to get around. It just takes a little bit longer and it’s a little bit more effort for the user. (Participant 3)	Add a side menu bar that is always available
