# Supplementary figures and images for: Using psychological theory and qualitative methods to develop a new evidence-based website about acupuncture for back pain
Source: Eur J Integr Med. 2016 Aug;8(4):384–93. doi: 10.1016/j.eujim.2016.05.006 (PMC5078494; doi:10.1016/j.eujim.2016.05.006)

Supplementary Material: Example Screenshots from Website


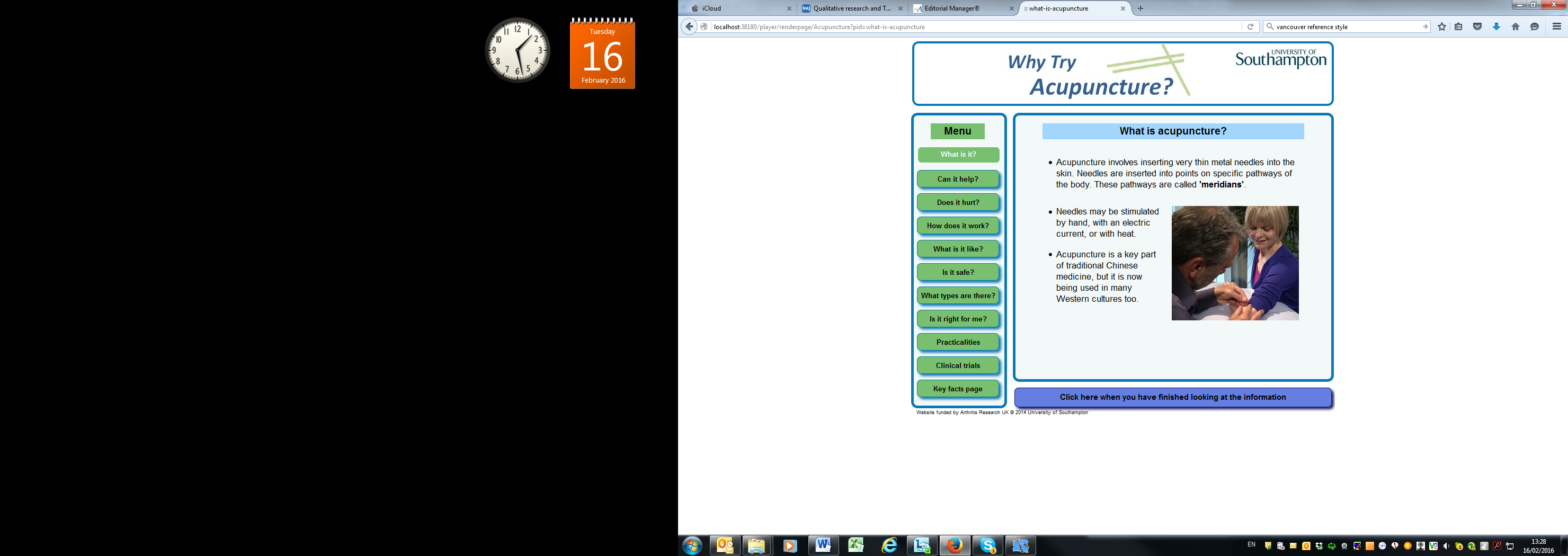


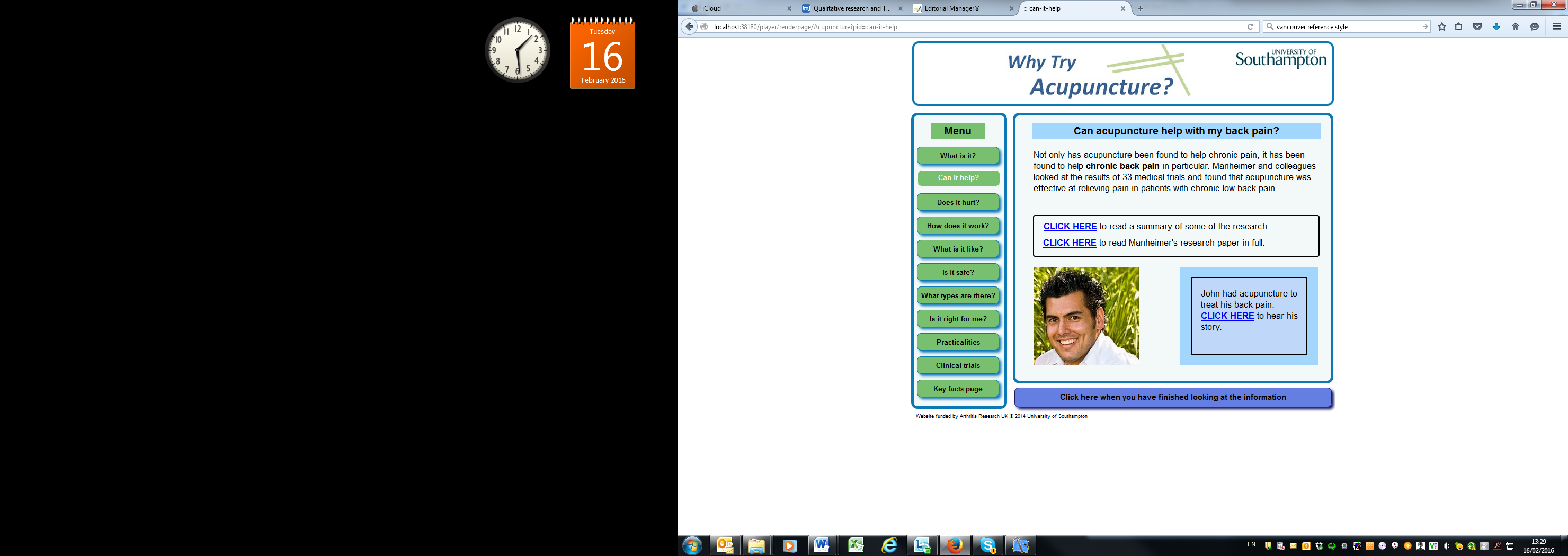


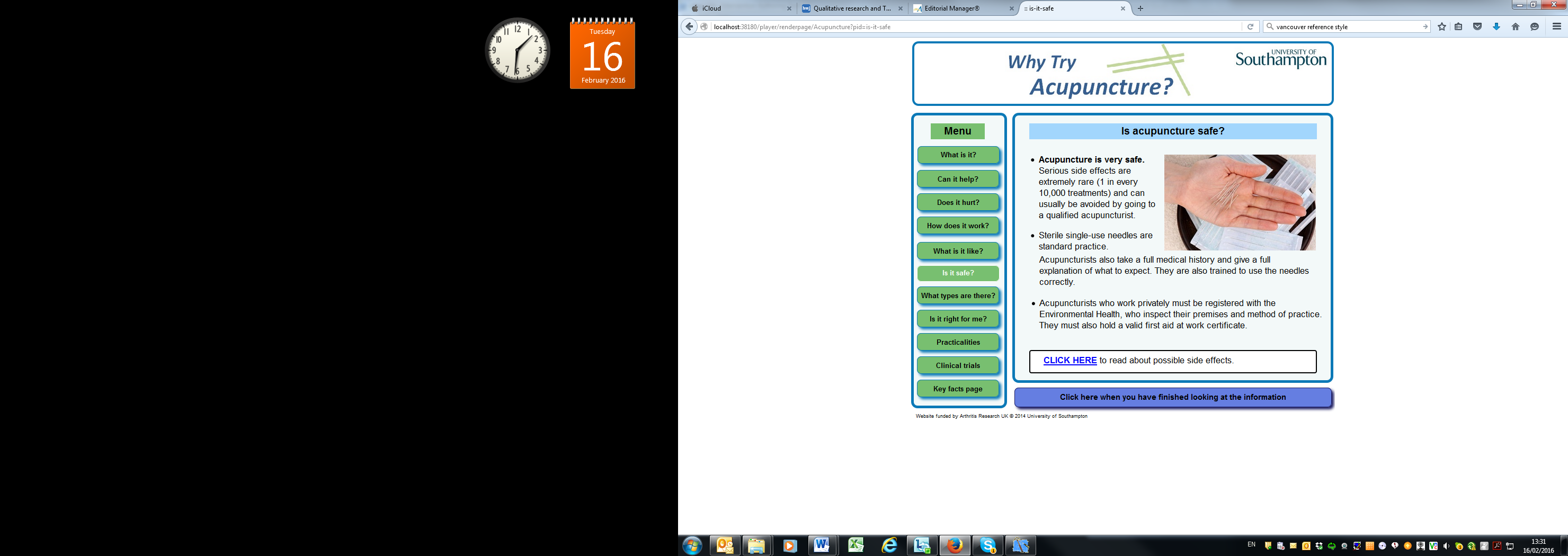


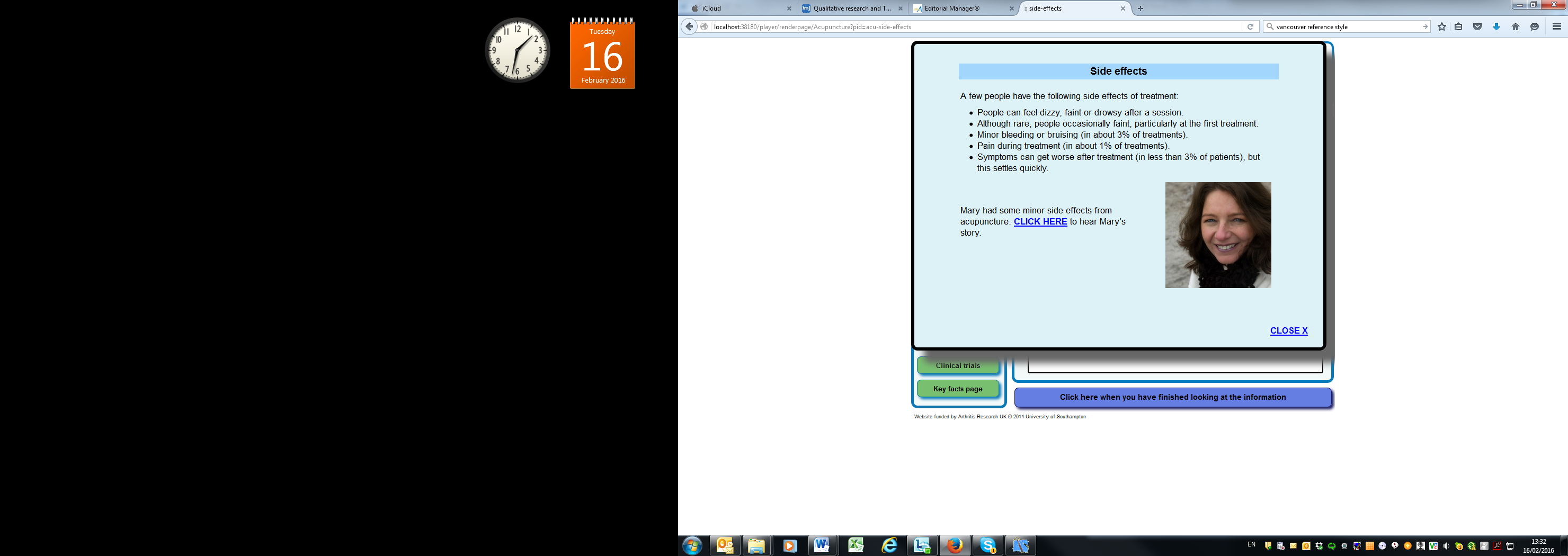

Supplement: Supplementary file 1 [file mmc1.docx]
